# Editorial: Cardio-Oncology: From Bench to Bedside

**DOI:** 10.3389/fcvm.2019.00037

**Published:** 2019-04-10

**Authors:** Jun-ichi Abe, Anil K. Sood, James F. Martin

**Affiliations:** ^1^University of Texas MD Anderson Cancer Center, Houston, TX, United States; ^2^Department of Molecular Physiology and Biophysics, Baylor College of Medicine, Houston, TX, United States

**Keywords:** cardio-oncology, onco-cardiology, cardio-toxicity, cardiovascular damages, anti cancer drugs

Although significant progress has been made by many investigators, arguably the field of cardio-oncology is still in early phases. We cannot create a field of cardio-oncology by just putting the cardiology and oncology in one box and mixing it. Only careful observations, patient care, thoughtful research, and enthusiasm can lead this field to the next step. Collaboration between oncologists and cardiologists is important, but the path to creating synergistic collaborations between oncologists and cardiologists has not been fully established. We believe that this is simply due to the lack of information, and additional platforms are needed to accelerate the communication between these disciplines and with other clinical and basic science groups. In this edition, we cover broad aspects related to the current practice and basic research of cardio-oncology.

Karlstaedt et al. proposed that by comparing metabolism in cancer cells and cardiomyocytes, we can obtain novel knowledge and therapeutic approaches for heart failure. Since it is thought that heart is the most “non-oncogenic” organ, this article reveals how important it is to learn cancer biology to fully understand cardiovascular disease.

The five review articles by clinician investigators provide an important message that clinical research based on diligent patient care and careful observations from the bed side form an essential part of cardio-oncology.

Fadol discusses the importance of the management of the cardiovascular complications of cancer therapy, which can significantly change the prognosis of chemotherapy-induced heart failure, and how important it is to generate evidence-based data to guide clinical decision making in the management of the cardiovascular complications of cancer therapy.

The remarkable progress of cancer therapy has led to a substantial increase in the number of childhood cancer survivors, many reaching childbearing age. This positive fact brings us a new and important question: can cardiac dysfunction be increased by pregnancy in women cancer survivors, especially among those treated by anthracyclines? Thompson's review provides an up-to date information about this important question and future goals to clarify this question.

It is well known that there is increased risk of venous and arterial thrombosis in cancer patients. Lee and Cameron discuss mechanism of thrombosis in cancer, and how to treat it. Liu et al. also describe the challenges for treating active coronary artery diseases in cancer patients, and provide strategy and guidelines for applying invasive cardiovascular procedures and intravascular imaging techniques to cancer patients. Lastly, Yusuf et al. have described the pathophysiology of radiation-induced cardiovascular disease. Although modern radiation therapy significantly minimizes the exposure of radiation to the heart, the dose of cardiac exposure to radiation remains high in many cancer patients.

We include six articles from the basic science side. Additional animal models to study the effect of cancer treatments on the vasculature are needed. Ko et al. have established a reliable mouse model that recapitulates radiation-induced cardiovascular disease in cancer patients. Three articles offer up-to date information regarding modern radiation therapy-induced cardiovascular dysfunction and its possible mechanisms. First, Sylvester et al. summarize the types of radiation currently in clinical use, and current knowledge of the mechanisms by which they cause cardiovascular disease. Second, Menezes et al. have discussed the therapeutic dose of radiation used in modern radiation therapy and its relationship to the cardiovascular disease. Lastly, Vu et al. proposed the potential role of the p90RSK-ERK5 module in regulating radiation-induced endothelial cell apoptosis.

Paez-Mayorga et al. have reported an interesting role of ERK5 SUMOylation, which can explain ponatinib-induced endothelial damage. This is a good example to illustrate the importance of communication between basic science and clinical information to define a new mechanism of cancer therapy-induced cardiovascular disease.

In the review by Dong and Chen the importance to understand cancer treatment toxicity against not only heart but also vasculature is summarized. Because of this, we need to broaden our interests from heart alone to the entire circulatory system as potential targets of cancer therapy-induced cardiovascular disease.

We believe that the topic of cardio-oncology from bench to bedside offers the opportunity for readers to know the challenges and potential future directions of cardio-oncology today, and we hope that more researchers, nurses, paramedical aides, and clinicians will appreciate this important and interesting, but still developing field of cardio-oncology.

## Author Contributions

JA has prepared the editorial with the consent from JM and AS.

### Conflict of Interest Statement

The authors declare that the research was conducted in the absence of any commercial or financial relationships that could be construed as a potential conflict of interest.

